# Metabolic Requirements for Spermatogonial Stem Cell Establishment and Maintenance In Vivo and In Vitro

**DOI:** 10.3390/ijms22041998

**Published:** 2021-02-18

**Authors:** Anna Laura Voigt, Shiama Thiageswaran, Nathalia de Lima e Martins Lara, Ina Dobrinski

**Affiliations:** Department of Comparative Biology and Experimental Medicine, Faculty of Veterinary Medicine, University of Calgary, Calgary, AB T2N 4N1, Canada; anna.voigt1@ucalgary.ca (A.L.V.); shiama.thiageswaran@ucalgary.ca (S.T.); nathalia.delimaemart@ucalgary.ca (N.d.L.e.M.L.)

**Keywords:** prepubertal development, spermatogonial maturation, spermatogonial culture, metabolism

## Abstract

The spermatogonial stem cell (SSC) is a unique adult stem cell that requires tight physiological regulation during development and adulthood. As the foundation of spermatogenesis, SSCs are a potential tool for the treatment of infertility. Understanding the factors that are necessary for lifelong maintenance of a SSC pool in vivo is essential for successful in vitro expansion and safe downstream clinical usage. This review focused on the current knowledge of prepubertal testicular development and germ cell metabolism in different species, and implications for translational medicine. The significance of metabolism for cell biology, stem cell integrity, and fate decisions is discussed in general and in the context of SSC in vivo maintenance, differentiation, and in vitro expansion.

## 1. Introduction

Spermatogonial stem cells (SSCs) are the basis of spermatogenesis and male fertility. SSCs can be isolated and transplanted, resulting in neospermatogenesis in the seminiferous tubules of germ cell-depleted recipients. SSC transplantation can serve as a powerful tool to generate transgenic animals for biomedical research, and potentially also for translational medicine and infertility treatment [1,2,3,4,5,6,7,8]. SSC development is characterized by the presence of distinct developmental embryonic and neonatal stages that are usually categorized by their specific tissue localization, making the SSCs a somewhat unique adult stem cell population [9,10,11,12,13].

Importantly, young male cancer patients are commonly treated with potent cytotoxic therapies and are at risk of irreversible damage to their immature sensitive germ cell pool, resulting in infertility. To allow these cancer survivors to father their own children in adulthood, prepubescent SSCs can be harvested and expanded in culture for later testicular re-introduction [1,2,3,4,5,6,7,8], which requires a detailed understanding of SSC developmental biology. Rodent prepubertal germ cells are widely used for experiments in male reproductive research. However, human reproductive development is characterized by the presence of an extended prepubertal period, in contrast to the rapid testicular maturation in rodents [14]. The study of human SSCs including characterization and development of clinical applications is limited by a practical deterrent. Many cells are necessary for effective experimentation and clinical use, but SSCs are sparse in the testis [15] and their in vitro expansion is necessary to amass the quantity of cells required for research. While culture systems enabling long-term SSC cultivation are established in rodents and smaller mammals, translation of these culture conditions do not yield the same results in larger mammals including humans.

The impact of metabolism on stem cells at distinct embryonic stages for both the maintenance of stemness and fate decisions has gained increasing attention in recent years, as demonstrated by an extensive list of reviews in the literature [16,17,18,19,20]. However, an understanding of the metabolic requirements and the importance of metabolism specific to SSC maturation, in vitro maintenance, and expansion, is only in its infancy [21,22,23]. However, it is pivotal to better understand the role of metabolism during the maturation of prepubertal human SSCs into adult SSCs to improve SSC culture systems for further clinical applications to treat infertility.

## 2. Development of the Male Gonad and Establishment of Spermatogonial Stem Cells (SSCs)

The mammalian testicular parenchyma is organized in seminiferous tubules and interstitial tissue. The tubules harbor Sertoli and germ cells and are enclosed by a peritubular myoid cell layer, while the interstitial tissue is composed of steroidogenic Leydig cells, blood and lymphatic vessels, connective tissue, and other cell types [24,25,26,27,28]. Regulation of the main testicular functions, sperm and androgen production (spermatogenesis and steroidogenesis, respectively), is complex and tightly controlled [24,29]. SSCs maintain the continuous sperm production required for male fertility [13,30,31,32]. SSC precursors originate from the embryonic bipotent primordial germ cells (PGCs) that migrate from the proximal epiblast to the gonadal ridge, where they are enclosed by Sertoli and peritubular myoid cells, forming the seminiferous cords [10,33,34,35].

Immature (fetal/neonatal) SSC precursors are commonly referred to as gonocytes or prospermatogonia, which are considered quiescent from the time of colonizing the seminiferous cords. This quiescence continues until they reenter the cell cycle, migrate to the basement membrane, and undergo maturation and differentiation, either to constitute the SSC pool or differentiate into spermatogonia that will later become sperm [36,37,38,39,40,41,42,43,44]. The transition from gonocyte to SSC is still poorly described, mostly due to the difficulty in unequivocally distinguishing these cell types from one another. However, recent data have shown that gonocyte fate may be defined by heterogeneous transcriptional and methylation signatures developed during quiescence [43,44,45,46,47].

The neonatal maturation of the testis in mammals is commonly characterized by an early testosterone peak. This neonatal testosterone surge in primates and humans is generally referred to as *mini puberty* [48,49] and contributes to the full maturation of the hypothalamic-pituitary axis [50]. The testosterone peak occurs just a few hours after birth in rodents, and after several months in higher mammals and humans [14,51]. It is associated with the movement of gonocytes to the basement membrane [52,53]. Hence, this migration toward the basement membrane occurs within a week after birth in rodents and can take up to nine months in humans [54,55].

**Figure 1:**
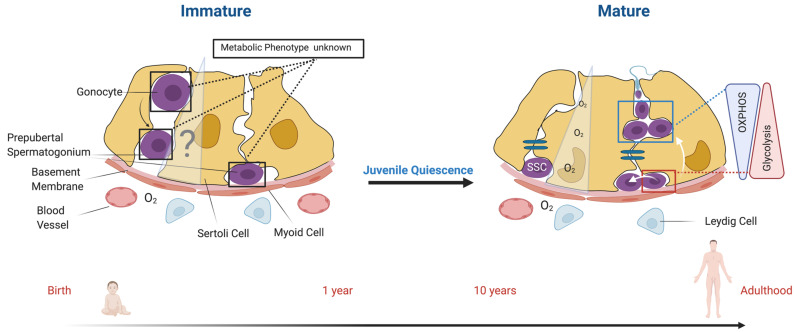
Schematic representation of the migration process and metabolic phenotype of human male germ cells during maturation (immature) and differentiation (mature). Immature (left; from birth to roughly 1 year in humans): Germ cells occupy distinct positions during immature spermatogonial development within the seminiferous epithelium. Gonocytes/prospermatogonia move from the center of the seminiferous cord to the basement membrane through a loose Sertoli cell scaffold. Metabolism of gonocytes/prospermatogonia and prepubertal spermatogonia, nutrient, and oxygen distribution in the immature seminiferous epithelium are unknown (question mark). Migrated spermatogonia reside in juvenile quiescence for over a decade before puberty. Mature (right; from puberty to adulthood): the Sertoli cells are mature and the seminiferous epithelium is compartmentalized by the Sertoli cell barrier as part of the blood testis barrier (blue). Within the adult testis, spermatogonial stem cells (SSCs) remain quiescent (single SSC), self-renew (white arrow at the basement membrane), or differentiate by migrating up to the adluminal compartment of the seminiferous epithelium (white arrow pointing up). SSCs rely on an anaerobic metabolism and switch toward oxidative phosphorylation (OXPHOS) with differentiation. The oxygen pressure decreases with diffusion through the epithelium. Elongated spermatid and sperm metabolism are highly complex and characterized by differences in metabolism between head and tail and therefore excluded from this simplified schematic (figure created with BioRender.com).

The gonocyte to spermatogonia transition is initiated prior to birth in mice. It was previously thought to be related to the movement of gonocytes from the center of the seminiferous cords to the basement membrane, but it is mainly guided by the crosstalk with Sertoli cells and intracellular activation of specific pathways. The transition is accompanied by the cytoplasmic-to-nuclear translocation of FOXO1, which requires fibroblast growth factor (FGF) signaling upstream of glial cell derived neurotrophic factor (GDNF) signaling and retinoic acid regulation [41,45,56,57,58]. Similarly in humans, PGCs—once migrated to the center of the developing seminiferous tubules—almost directly mature to the transcriptional landscape of an adult undifferentiated spermatogonial stage prior to birth [59]. However, their migration to the basement membrane of the tubules does not occur until several months after birth. Some studies in higher mammals and humans have reported some spermatogonial heterogeneity neonatally and the appearance of differentiating spermatogonia prior to puberty [39,60,61,62,63]. While SSC precursors acquire the transcriptional profile of adult spermatogonia rapidly during human embryogenesis [59], it remains to be determined if these cells are functionally or biochemically distinct from adult SSCs during their change in localization and juvenile quiescence (see Figure 1), similar to what we recently described for porcine spermatogonia [64].

Adult SSCs are a rare type of undifferentiated spermatogonia which comprise around 0.03% of the total germ cells in mice. This percentage may be slightly higher in nonhuman primates and humans [15,65]. SSCs are usually located in a distinct position inside the seminiferous epithelium, referred to as the spermatogonial stem cell niche [66,67,68,69,70,71]. Within this microenvironment, cytokines, growth factors, an6d intercellular contacts precisely regulate SSC fate. SSCs within their niche either self-renew, remain quiescent, or generate spermatogonia committed to differentiation [28,32,66,67,68,72,73]. Regulation of the niche microenvironment is complex and involves contributions of Sertoli cells, peritubular myoid cells, the basement membrane, macrophages, and the vascular network, while Leydig cells may be more involved in stimulating spermatogonial differentiation [28,31,68,70,71,72,74,75,76,77]. Metabolic regulation plays a central role for regulation of numerous cellular events, but its influence on SSC development has yet to be investigated.

Establishment of the SSC niche may also be related to the quiescent/active state of SSCs, which differs according to the species and timing of development [28,78,79]. Some variation in niche regulation has been described between neonatal ages and adulthood in mice, but testis development in laboratory rodents happens very quickly, with the first wave of spermatogenesis starting around postnatal day 8 and being completed at postnatal days 30–35 [39,80,81,82]. This lack of prepubertal quiescence in rodents makes it difficult to identify specific stages [28,83]. In contrast, more than a decade is required for prepubertal testis development and gonadal maturation in higher mammals and humans, generally characterized by the existence of a juvenile pause and an extended time span of prepubertal development [60,81,84,85,86]. Testicular tissue reactivation at the end of puberty, called *gonadarche*, occurs between 9–13 years in human [50], and after several years in most non-human primate species [87,88,89,90,91,92,93]. Before *gonardache,* there is a period of gonadal dormancy, characterized by low gonadotropin secretion, minimal testosterone secretion, discontinued Sertoli cell proliferation, and variable mitotic activity of germ cells in primates and human [94,95,96,97,98,99,100,101].

Immature SSC precursors have lower transplantation efficiency than more mature SSCs, which may be generally related to different cellular metabolism depending on the stage of development [12,60,81,102,103,104]. Recent studies using single cell RNA-Seq analysis have identified different spermatogonial subsets/states during human testis development, confirming previous descriptions of a heterogeneous protein expression profile [36,38,47,61,62,68,105,106,107,108,109]. Considering the discrete developmental stages observed during SSC development, coinciding with a distinct tissue localization and the differences in reproductive physiology between rodents and higher mammals, it is highly likely that metabolically “immature” SSC precursors are existent in the testis of higher mammals and humans for an extended period of time. This requires careful attention for laboratory handling and subsequent clinical usage.

## 3. The Current State of Germ Cell Culture

It has been almost two decades since mammalian germ cell culture was initially established, validated by the continuous expansion of mouse spermatogonia in culture with subsequent successful transplantation and recovery of spermatogenesis in recipient testis lacking endogenous germ cells [110,111,112]. The combination of specific cytokines, SSC enrichment, and use of feeder layers provide the necessary microenvironment to expand mouse spermatogonia in vitro. Glial cell derived neurotrophic factor (GDNF) regulates SSC self-renewal in vivo [113,114] and mouse SSC expansion in culture was achieved when GDNF was added to media, clearly demonstrating its crucial role for SSC maintenance in vivo and in vitro [110,112]. GDNF promotes SSC self-renewal through the activation of, and cross-talk with multiple pathways [115,116], highlighting the complexity of the process. In addition to GDNF, other cytokines such as basic fibroblast growth factor (bFGF), epidermal growth factor (EGF), and soluble GDNF family receptor alpha (GFRα), among others, have been identified as factors that support mouse SSC renewal [111,112]. 

Various media formulations have since been developed for rodent germ cell culture using alphaMEM [112] or StemPro-34 [110] as the basal medium, with varying amounts and combinations of growth factors, serum, albumin, essential amino/fatty acids, vitamins, insulin, putrescine, transferrin, biotin, 2-mercaptoethanol, and HEPES buffer [117], for example. Of note, media supplementation with GDNF alone is sufficient for the expansion of spermatogonia derived from some mouse strains, while others require additional synergistic self-renewing pathway activation by other growth factors. For example, when cultured in serum-free media including GDNF, SSCs isolated from mice with DBA/2J-background were able to proliferate while SSCs isolated from the C57BL/6-background required the addition of bFGF and soluble GFRα to efficiently activate the GDNF pathway [111].

Furthermore, besides GDNF, Sertoli cells and other testicular somatic cells produce growth factors that are implicated in both SSC self-renewal and differentiation [112,118,119,120]. A high contamination with somatic cells makes culture conditions consequently less defined [117]. Additionally, differentiating spermatogonia have an inhibitory effect on the expansion of their undifferentiated counterparts [121,122,123]. Therefore, isolating cells from prepubertal animals provides an advantage due to the absence of differentiated cells, resulting in a relatively higher number of undifferentiated spermatogonia obtained. Somatic cells from immature tissue, however, proliferate quickly, overgrowing germ cells in culture [110,124]. Therefore, it is important to ensure a highly enriched starting population prior to introducing spermatogonia to culture, so that the potential detrimental effects of contaminating cell types can be minimized.

Interestingly, the controlled introduction of additional cells as feeder layers has been shown to be supportive for SSC expansion. Feeder cells consist of mitotically arrested, adherent cells that act as a substrate and are capable of producing and secreting factors to condition media in which cultivated cells, like SSCs, can grow [125]. The feeder layer can mimic the niche microenvironment, a multifaceted metabolic, biochemical, and physical niche that supports the distinct stem cell metabolism [126,127,128,129]. Aside from the advantages such as growth factor production and serving as a scaffold for proliferating cells [125], many biochemical and molecular mechanisms underlying the benefits of feeder layers are still largely unknown. While the feeder layer has been identified as beneficial for proliferation and maintenance of stem cell activity [130], it adds a level of variability and potential contamination due to insufficient enrichment after collection, making clinical applications problematic. Additionally, the presence of feeders may rapidly change the cellular metabolic flux, which is extremely sensitive and has wide-ranging consequences for the cultured cells. For SSC experimentation and clinical translation, it is therefore desirable to establish highly defined, feeder- and serum-free culture conditions. 

A variety of culture conditions and media formulations have been derived for germ cells from mice, other rodents, and small mammals [110,131,132,133,134,135]. However, applying these conditions to expand spermatogonia isolated from larger animals has met with a lesser degree of success, demonstrated by relatively shorter proliferative periods and/or lower maintenance of stem cell activity [136,137,138]. The failure to culture SSCs from higher mammals may be related to insufficient activation of the GDNF pathway, similar to what was described for different mouse strains [111]. Pronounced GDNF pathway activation increases glycolytic flux [111,139], which demonstrates an interaction of SSC cell fate regulation and metabolism, suggesting a mismatch between culture conditions and the metabolic requirements of SSCs at variable developmental stages of maturation. Recent studies of rodent SSC culture [23] identified mainly progenitor cell expansion with a continuous decline of stem cell number [110,111,140,141,142], accompanied by a lower transplantation efficiency proportional to their time in culture [21,23]. 

While the majority of media formulations and culture conditions used for the expansion of SSCs promote mitochondrial respiration [111,112,117,142,143], a recent study has shown that these conditions might actually be detrimental for stem cell maintenance [23]. Interestingly, prevention of rapid loss of stemness or prolonged maintenance of stem cells in culture can be achieved by glycolysis activation in distinct culture conditions [23,139]. While transcriptional regulation and enzyme abundance are critical for metabolic regulation, they are generally overestimated in their role for fast changes in metabolic flux [144]. Allosteric regulation makes metabolism extremely sensitive to nutrient and oxygen availability and their fluctuations, and these have to be tightly controlled in defined in vitro culture systems [145]. Any culture condition alternating metabolic states applies a stochiometric pressure causing fast metabolic transitions, pushing a cascade of wide-ranging cellular changes [146,147,148]. Therefore, gathering more information about different maturational stages and metabolic profiles of spermatogonia, especially immature SSCs, will inform more specific laboratory handling practices to enable successful in vitro expansion of functional SSCs.

## 4. Stem Cell Metabolism

Stem cells are characterized by their capability to self-renew and regenerate tissue, which requires responsiveness to external and internal cues to allow rapid adaptation for the maintenance of tissue function [149]. Such sensitivity requires a high degree of metabolic plasticity [150]. For a long time, metabolism was described in a simplified fashion as the catabolism of nutrients that yield energy in the form of adenosine triphosphate (ATP) [151]. However, the impact of metabolism goes beyond the production of energy encompassing growth, regulation of lineage commitment, epigenetic control of stem cell maintenance [152], and influencing pathway activation [139]. 

In stem cell research, the effects of mitochondrial or non-mitochondrial metabolism on cell fate have been studied. Here, non-mitochondrial (anaerobic) metabolism refers to the simple enzymatic degradation of glucose to lactate within the cytoplasm, which does not require oxygen (although it might be sufficiently available in some cases, as discussed later). Mitochondrial (aerobic) metabolism refers to mitochondrial oxidative decarboxylation and subsequent oxidative phosphorylation (OXPHOS), either from carbon compounds produced during glycolysis or from the direct oxidation of pyruvate/lactate or fatty acids. OXPHOS requires oxygen and yields approximately 36 molecules of ATP per glucose molecule [153]. This process utilizes a highly complex electron transfer system along the mitochondrial membrane, which is sensitive to malfunction and is accompanied by the continuous production of potentially harmful reactive oxygen species (ROS) [154,155].

Various adult stem cells have been described as having low mitochondrial respiration and performing anaerobic metabolism [129,156,157,158,159,160,161,162] prior to shifting toward OXPHOS during differentiation [47,61,157,163,164,165]. This would suggest that anaerobic metabolism is preferred for stem cells, whose lifelong genomic integrity is crucial for tissue maintenance. Conversely, after fertilization, the totipotent zygote has the potential to form the whole organism. The preimplantation embryo has been described to rely on pyruvate consumption, demonstrating a preference for highly oxidative metabolism [166,167], while a switch to anaerobic metabolism has been observed with further development [151,168,169,170]. These transitions are described in several species, seem to be required for stem cell integrity and fate decisions [152,171], and are accompanied by individual cellular activities and developmental stages [150,171,172,173]. Therefore, specific culture conditions favoring distinct metabolic flux can be utilized to support reprogramming events [174,175,176,177,178]_._

As metabolism is highly variable in embryonic stem cells and adult stem cells, depending on their biological activity, qualifying stem cell metabolism as either mitochondrial or non-mitochondrial is limiting. Rather, cellular metabolism is characterized by variable combinations of metabolic pathways to perfectly fit redox homeostatic and metabolic requirements for stem cell maintenance or lineage specification. In this context, metabolism can be characterized by highly glycolytic flux at the same time as some mitochondrial metabolic pathways are active to meet specific cellular demands [150,152]. However, some questions arise: Why do cells in distinct developmental stages prefer different metabolic states? and what does this tell us about the biology of the cell of interest?

### 4.1. Metabolism during Proliferation

Proliferating cells require biological building blocks such as lipids, proteins, nucleotides, and reductive cofactors. The interesting phenomenon that highly proliferative cells mainly perform glycolytic catabolism from glucose to lactate, despite having sufficient oxygen availability to complete the more energy efficient oxidative phosphorylation, was first described by Otto Warburg (the Warburg Effect) [179,180]. 

While this metabolic phenotype initially seems inefficient, glycolytic flux is rapid and can quickly surpass the ATP production of respiration [181]. High glycolytic flux without complete metabolism of carbon compounds within the mitochondrion is advantageous for a proliferating cell [150]. It yields an accumulation of glycolytic intermediates, which can be fed into the pentose phosphate pathway (PPP) for the production of metabolites and reductive cofactors for anabolic processes [175,182]. 

### 4.2. Redox Homeostasis 

ROS are highly unstable oxygen compounds, mainly generated as a by-product during mitochondrial respiration [154] and are produced by cytoplasmic oxidases [183]. Their important cellular roles have long been overlooked, overshadowed by the detrimental consequences of oxidative damage for cellular longevity and genomic integrity [183,184]. Besides cellular ROS, reductive cofactors or coenzymes such as nicotinamide/flavin adenine dinucleotide compounds, produced during different metabolic pathways, contribute to a cellular redox status required for different pathways. 

Cellular pathways are extremely sensitive to a changing redox balance through oxidation and reduction dependent protein activities [183]. Especially in stem cells, this cellular fine tuning makes cells extremely susceptible to rapid reactions and the correct balance is critical for nucleo-mitochondrial communication [128,185]. Various nuclear processes like transcription, epigenetic changes, and the cell cycle are coordinated by cellular metabolism influencing the redox balance [184]. 

### 4.3. Control of the Epigenetic Landscape

Epigenetic programming is a hallmark of cellular specification. Fate decisions during development are controlled by the crosstalk between epigenetic modifiers and cell fate determining transcription factors [186]. Acquisition of a specific epigenetic and transcriptional landscape has been depicted as a ball rolling down a hill through branching canals into a valley, the so called Waddington’s canal [186,187]. In this depiction, the ball reaching the valley represents lineage specification and illustrates the gain of stability at the cost of general plasticity. Epigenetic modifications are controlled and maintained by specific enzymes that cause chromatin remodeling through DNA methylation and histone modifications (acetylation, phosphorylation, ubiquitylation, and methylation among others), and contribute to the transcriptional profile by varying genomic accessibility independent of the genomic sequence [186]. These epigenetic modifiers often require coenzymes and are therefore dependent on and are highly sensitive to the availability of metabolic intermediates and reductive cofactors from glycolysis, tricarboxylic acid cycle (TCA), OXPHOS, and amino acid metabolism [188,189,190,191]. Consequently, metabolic and redox fluctuations and status directly influence chromatin remodeling in undifferentiated and differentiated cells, and therefore control cell fate [152,188,192,193,194,195].

In summary, metabolism and its changes are implicated in wide-ranging cellular processes. The reliance on different metabolic pathways plays a central role in cellular homeostasis, but also maintenance and lineage commitment of different stem cells. The establishment of a defined but supportive and physiological culture condition that meets the stem cell specific metabolic requirements remains one of the biggest challenges in stem cell research. This underlines the importance of understanding and reproducing the metabolic niche to achieve the physiological expansion of SSCs in vitro.

## 5. Metabolic Regulation within the Testis

### 5.1. The Metabolic Spermatogonial Stem Cell (SSC) Niche

Spermatogonial stem cells reside at the basement membrane of the seminiferous tubule among single spermatogonia (the ‘A single’ model reviewed elsewhere) [26,27,28,119,196,197], and are regulated by their niche and its interaction with intrinsic cellular regulatory circuits. These single spermatogonia are highly heterogenous and, interestingly, even spermatogonia that are more committed to differentiation can act as SSCs depending on specific environmental cues [198,199,200]. While a stem cell niche is generally characterized by a combination of specific cellular interactions, extracellular matrices, and growth factors, it also has a certain ‘stem cell-specific’ metabolic environment, a *metabolic niche.* Recent studies have shown that this metabolic regulation is pivotal for SSC maintenance in vivo and *vitro* [21,23,139]. However, besides Sertoli cell metabolism (discussed later), the metabolic phenotype of the majority of niche cells remains to be investigated and therefore the detailed involvement of the interstitial compartment in the specific SSC metabolic environment is still unclear.

The precise localization of the SSC niche is still under debate [22,198,201]. Several studies suggest that true SSCs are closely associated with the vasculature [66,68,202]. The purported SSC population is dispersed among differentiating spermatogonia [68,203] in a so called *open niche* model [198]. However, the majority of stem cells in the body are described to reside in a *closed niche*, where regulatory factors are relatively easy to define and explain, while investigation of *open* or *facultative* niche models is ongoing [149,203]. A revised view of the common A single model suggested that SSCs do not reside close to the vasculature [22,201], but rather in a hypoxic niche, similar to other adult stem cells [128,129]. The seminiferous tubule has a significantly lower oxygen partial pressure than the interstitial tissue [204,205,206], with a declining oxygen gradient from the basement membrane to the lumen [205] (see Figure 1). Similar to oxygen, nutrients require diffusion to reach the seminiferous tubule lumen. Therefore, not only spermatogenesis, but also maturation occurs along an oxygen and nutrient gradient.

### 5.2. The Role of Sertoli Cell Metabolism

For a long time, a metabolic teamwork has been described in the seminiferous tubules between nursing, highly glycolytic Sertoli cells [146,207,208] and the lactate consuming differentiating germ cells [209,210,211,212]. Sertoli cells compromise a large part of the SSC niche and not only contribute with an immunoprotected scaffold and cytokines, which are crucial for spermatogenesis [27,119], but also create a metabolic microenvironment that plays a role for sufficient spermatogenesis [146].

Sertoli cells are an intriguing differentiated cell type as they, very different from other specialized cell types, exhibit high glycolytic flux despite sufficient oxygen availability (Warburg effect) [179,180,207,208,213]. This high glycolytic flux occurs during development, coinciding with high proliferation of immature Sertoli cells, but persists during adulthood [146]. The various physical, endocrine, and biochemical functions of adult Sertoli cells, even though terminally differentiated and non-proliferative, requires a tremendous build-up of biomass and its constant turnover throughout male lifetime to support spermatogenesis [153]. Highly glycolytic flux and reduction of pyruvate can, therefore, serve two-fold: directly providing the metabolic environment (high lactate and pyruvate concentration in the seminiferous tubule), and forming the continuously changing physical/paracrine environment by using the glycolytic carbon intermediates in anabolic pathways instead of oxidative decarboxylation for subsequent oxidative phosphorylation and ATP production [214].

In comparison to the adult Sertoli cells that form strong intercellular junctions that compose the blood–testes barrier [146], immature Sertoli cells display a loose scaffold and accompany immature SSC precursors in this basic niche [60] on their way to the basement membrane. During development, the SSC niche is being established along with niche maturation events and the seminiferous tubule compartmentalization. Developing germ cells are therefore exposed to maturational changes in Sertoli cells, which might have an impact on nutrient distribution within the emerging seminiferous epithelium (see Figure 1). As Sertoli cells remain highly glycolytic during their maturation, germ cells are exposed to high amounts of lactate produced and excreted by Sertoli cells throughout development and steady-state spermatogenesis [146,207]. Besides serving as a nutrient, lactate may play a role in SSC maturation. Similarly to cancer cells, in which lactate was shown to enhance mTOR activity [215], it also contributes to the activation of AKT [216], inhibits apoptosis [217], and enhances transcription and translation through-put [218] in male germ cells. 

## 6. Metabolism of Male Germ Cells

Germ cells have a pivotal role in development by transmitting genetic information to the next generation. During germ cell development, epigenetic marks are erased and subsequently re-established during gametogenesis [187,219]. 

Undifferentiated spermatogonia, which are at the basement membrane of the adult seminiferous epithelium with access to blood-derived glucose, have long been suggested to rely on anaerobic metabolism [211,220]. Recent transcriptomic profiling of testicular cells confirms this hypothesis by showing an enrichment of glycolysis-related genes in undifferentiated spermatogonia/SSCs and an upregulation of OXPHOS linked genes during differentiation [47,62,221,222]. Just recently, more attention has been given to the metabolism and metabolic changes during culture and its importance for mouse stem cell maintenance [21,23]. Only two functional studies on SSC metabolism have been performed to date, demonstrating a beneficial effect of anaerobic glycolysis on SSC function [23,139]. Importantly, at the same time, protection of SSC integrity requires preservation of mitochondrial function [21] in preparation for later differentiation [221], for which a shift toward OXPHOS is paramount [166]. With differentiation, male germ cells migrate toward the lumen of the seminiferous epithelium for subsequent release as spermatozoa. The change in their microenvironment during differentiation is accompanied by epigenetic changes and several metabolic transitions [47,61,223,224,225]. Spermatocytes and round spermatids consume pyruvate and lactate [218,226,227] that are interconvertible, accompanied by a change of redox balance and the production of NAD^+^ or NADH, respectively. It seems that the preferential lactate consumption of spermatocytes and round spermatids [209,212,218] contributes to redox homeostasis, determining differentiating germ cell function. Lactate is required for RNA and protein synthesis [218,228], and for energetic maintenance of round spermatids [210,212,226]. Interestingly, elongated spermatids and spermatozoa seem to undergo, at least partly, a transition toward glycolysis [229,230,231,232,233]. Hyperacetylation is required for the histone to protamine exchange in developing spermatozoa to allow for tight chromatin compaction [224,234,235], which facilitates this metabolic transition [225]. The correct histone to protamine transition can be impaired by environmental toxins [236], which therefore could indirectly impair the metabolic performance of male gametes and therefore fertility. This aspect requires further investigation.

### Regulation of Metabolic Transitions in the Germline

As the seminiferous tubule presents a gradient based metabolic microenvironment, changes in nutrient availability and oxygen partial pressure could trigger or support the germ cell transition processes. In mouse SSCs, low mTOR activity is essential for stem cell maintenance [237,238]. This low activity is also required to maintain high autophagic flux for glycolytic metabolism in hematopoietic stem cells (HSCs) [239]. Upregulation of mTOR is associated with differentiation of spermatogonia [240,241,242] and these activity changes are likely controlled by distinct transcriptional regulatory circuits [237]. 

Glyceraldehyde phosphate dehydrogenase (GAPDH) seems to play an essential role for glycolysis regulation in the male germ line. GAPDH regulates glucose intake in spermatocytes [226], glycolysis in spermatids [243], and glucose consumption in spermatozoa [244,245]. Noteworthy, GAPDH is routinely used as a housekeeping gene or protein, however, its abundance is highly variable with different metabolic stages, especially in germ cells and, as such, should be carefully evaluated depending on the experimental design. 

Fast metabolic changes in the germline are enabled by translational regulation [246,247]. This is most likely coordinated by the germ cell specific nuage, a ribonucleoprotein dense structure found in the cytoplasm [248,249,250,251]. This translational control allows for fast and efficient changes during spermatogenesis and can make transcriptional analysis misleading with regard to metabolic flux events.

## 7. Missing Information and Perspectives

As discussed earlier, testicular maturation events occur rapidly in rodents, while taking weeks/months in the majority of higher mammals [252] and several years in humans [253]. The transition of gonocytes to SSCs is traditionally seen as the movement of the cell toward the basement membrane, but has been implicated to start prior to birth by the activation of SSC specific pathways in mouse [45] and the early embryonic establishment of the transcriptional profile of adult spermatogonia in human [59]. However, the exact mechanisms and characteristics of this early germ cell maturation are still unclear. In combination with the existence of the juvenile pause in higher mammals and humans, the metabolic and physiological stages of neonatal and prepubertal spermatogonia in humans and most higher mammals are ill defined (see Figure 1). 

PGCs rely on mitochondrial respiration, which is required for extensive epigenetic changes [196,254,255]. Early prepubertal pig (1-week-old) and human (<1-year-old) spermatogonia have high ultrastructural similarity and are characterized by thick round perinuclear accumulated mitochondria. In contrast to prepubertal mouse, early prepubertal pig spermatogonia (1-week-old) rely on OXPHOS fuelled by the oxidative decarboxylation of pyruvate, which is accompanied with high resistance to ROS [64]. As described above, SSCs rely on anaerobic metabolism, raising the question of when this metabolic transition occurs in human. In pig, the beginning of metabolic transition toward an anaerobic metabolism could be detected at two months of age [64]. Interestingly, recent single cell RNA profiling of human testis could not detect metabolic changes from human PGCs to adult undifferentiated spermatogonia [59]. The metabolism of gonocytes has, however, not yet been described in human and other higher mammals except for the pig [64]. Just recently, it was shown that rat gonocytes exhibit an extensive antioxidative machinery [256], and due to their distinct position within the seminiferous tubule, it seems likely that rodent and higher mammalian maturing germ cells undergo similar transitions during development as they do during spermatogenesis, albeit at a different pace. 

Some cancer patients are very young at the time of treatment, and it is likely that metabolically immature SSC precursors are present in their testis for a long time. Therefore, it is essential to use animal models that display a similar timescale of testis development and juvenile quiescence as humans. Precocial higher mammal models combine the existence of a juvenile pause with compressed reproductive development [51], which allows for more detailed studies of developmental reproductive biology. 

As the metabolism of gonocytes has not been investigated and characteristics of gonocyte maturation are still under debate, the exact time point and the metabolic fingerprint of distinct developmental germ cell stages remain to be elucidated. Single cell transcriptomics allows for the profiling and screening of cell specific characteristics during tissue development [59]. However, especially in the germline, various metabolic enzymes are already transcribed to prepare the cell for rapid cellular changes albeit being blocked for translation [246]. Therefore, the combination of epigenetic and metabolomic profiling depicts a broad landscape for biomarker discovery for stem cells and differentiating cells [257]. 

How do we apply the knowledge on metabolism to laboratory handling? It has become clear that mouse SSCs are more resistant to metabolic changes and able to persist in culture for an extended period of time, although the conditions still need to be optimized. Apparently, adult SSCs require glycolysis optimized conditions, characterized by low oxygen and high glucose to maintain SSC integrity [23]. Additionally, long term culture of SSCs under conventional conditions result in mitochondrial damage, with a compensatory glycolysis dependence and loss of differentiation competence [21]. These studies underline the detrimental effects of non-physiological metabolism on SSC maintenance in culture and the importance to adapt culture conditions to the metabolic needs of stem cells and to protect metabolic plasticity, needed for differentiation. 

The solution, however, might be more complex for immature SSC precursors or gonocytes, which potentially rely on a different metabolism than adult cells for an extended period of time in most higher mammals and human [64]. Gonocytes may require time for physiological maturation, and accelerating maturation in vitro might have detrimental effects. It is also not clear if immature SSC precursors can successfully adapt to adult SSC culture conditions. Future research will increase our understanding about basic metabolic requirements for SSC maturation and integrity, and how SSC metabolism is established and protected within the niche. This knowledge will inform defined culture conditions that closely mimic this complex microenvironment.

## Data Availability

No new data were created or analyzed in this study. Data sharing is not applicable to this article.
